# A comparative study of total quality management of health care system in India and Iran

**DOI:** 10.1186/1756-0500-4-566

**Published:** 2011-12-28

**Authors:** Ali Morad Heidari Gorji, Jamal A Farooquie

**Affiliations:** 1 Department of Business Administration, Aligarh Muslim University (AMU), Aligarh, India

## Abstract

**Background:**

Total quality management (TQM) has a great potential to address quality problems in a wide range of industries and improve the organizational performance. The growing need to take initiatives by hospitals in countries like India and Iran to improve the service quality and reduce wastage of resources has inspired the authors to develop a survey instrument to measure health care quality and performance in the two countries.

**Methods:**

Based on the Baldrige health care criteria for performance excellence 2009-2010 and the guidelines proposed by the American Hospitals Association for hospitals in pursuit of excellence, compared health care services in three countries. The data are collected from the capital cities and their nearby places in India and Iran. Using ANOVAs, three groups in quality planning and performance have been compared.

**Result:**

Results showed there is significantly difference between groups and in no case the hospitals from India and Iran are found scoring close to the benchmarks. The average scores of Indian and Iranian hospitals on different constructs of the IHCQPM model are compared with the major results achieved by the recipients of the MBNQ award.

**Conclusion:**

In no case the hospitals from India and Iran are found scoring close to the benchmarks (Baldrige health care criteria for performance excellence 2009-2010 and the guidelines proposed by the American Hospitals Association for hospitals). These results suggested to health care services more attempt to achieve high quality in management and performance.

## Background

Good health, responsiveness to the expectations of its people, and financial contribution to the nation are the goals for health care systems of a country [1]. An overview of the health scenario all over the world indicates that despite having numerous excellent health care facilities, there exists a sufficiently large gap between the demand and delivery. In India nearly 1 million people die every year due to inadequate health care and two-third population is deprived of specialist care. The global health observatory [2]. Also reports a per capita expenditure of US$ 215 on health in Iran. There are nine physicians working for every 10, 000 persons in Iran.

With increasing competition, advances in medical sciences, and rising patient expectations, the health care systems have become complex organizations. They need to obtain an optimum balance between the resources and patient satisfaction. Total quality management (TQM) has a great potential to address quality problems in a wide range of industries and improve the organizational performance [3,4]. Juran (1995) has defined TQM as the system of activities directed at achieving delighted customers, empowered employees, higher revenues, and reduced costs. It is a philosophy aimed at continuously improving the quality and process to achieve customer satisfaction[5]. Simply stated, it is the building of quality into products and process making quality a concern and responsibility for everyone in the organization [6]. The term organizational excellence appears to be used as a synonym of business excellence in the quality-related literature. Tracing the evolution of organizational excellence as a concept, McAdam [6] reports that activities directed towards organisational excellence gained momentum in the early 1990s after the advent of quality awards like European Quality Award and Malcolm Baldrige Award. This has been defined as a key stage on the TQM journey and measures the effectiveness of TQM implementation [6]. Kelley and Hurst [7], in their project on health care quality indicators, refer to the manual of Organisation for Economic Cooperation and Development (OECD) and Institute of Medicine (IOM) to define the quality of health care as "the degree to which health services for individuals and populations increase the likelihood of desired health outcomes and are consistent with current professional knowledge". The Malcolm Baldrige criteria for performance excellence in health care organizations define [7] performance excellence as an integrated approach to organizational performance management that results in (a) delivery of ever-improving value to patients and stakeholders, contributing to improved health care quality and organizational sustainability, (b) improvement of overall organizational effectiveness and capabilities as a health care provider, and (c) organizational and personal learning.

Studies have suggested quite a large number of factors/elements/constructs/dimensions of TQM implementation. Many of them have appeared more frequently than others. TQM and performance improvement have a positive relationship, particularly, the Malcolm Badrige quality award criteria confirms such relationship between quality management practices and business results.

A study by Salaheldin (2009) indicates that there are many empirical studies which examine TQM practices-performance relationships in large firms but the small and medium firms still need a little more attention of researchers[8]. While the literature concerning service quality dimensions in the healthcare industry is replete with studies from the developed world, researchers from developing countries have been exploring the applicability of the related models and frameworks in their specific context. In Indian context, there is a dearth of an independent model of service quality as almost all the existing studies applied SERVQUAL framework, except that of Duggirala et al. [9,10]. Iran too does not seem to have any established framework for measuring quality efforts and performance of its health care industry.

Zakuan et al. [4] suggest that despite the number of publications and quantity of research on TQM, there is actually little empirical work that has been carried out in developing countries, particularly in the ASEAN region. Though there are evidences of recent studies in India and Iran pertaining to total quality management and performance in health care [9-13], none of them claims for having addressed the issue in totality. The current state of research in the area of health care quality along with the inadequacy and cost of health care services in India and Iran [2] seem to justify the present study entitled "A Comparative Study of TQM Practices in India and Iran". The purpose of including the United States in this study is to learn from their experiences and benchmark the Indian and Iranian services against those in the United States.

## Methods

The primary data are collected from India and Iran. In India, the researcher has contacted the Ministry of Health and Family Welfare in New Delhi seeking its permission and requesting the support required for this purpose. With the help of this office, 43 hospitals from all over the capital city and representing the government, semi-government, private, small, medium, and large types were initially contacted on convenience basis. The contact persons were mainly administrators and mangers. 32 responses could be obtained from this city. Another 25 hospitals were approached for data collection in Aligarh, a district headquarter in the state of Uttar Pradesh, where the researcher is pursuing the work at the Aligarh Muslim University. These hospitals were identified using an official directory and the basis of including them in the sample has again been the convenience sampling method. 21 respondents completed the questionnaire. A similar procedure was adopted in Iran to collect data from the capital city, Tehran and the state Mazandaran, the researcher's home town. After scrutinizing and editing the filled-in questionnaires, 110 were finally complied for further processing. Out of which, 50 are from India and the remaining 60 from Iran. Consent obtained from all participated. Prior to the actual collection of data, a pilot survey was done in Aligarh to judge the suitability of the questionnaire. For Iranian respondents, the questionnaire was translated in Persian to make it more compatible with their system. The translated questionnaire was first tested for its validity using a pilot study of 10 experts.

In addition to the information gathered through literature survey, two documents, namely, guidelines for hospitals in pursuit of excellence [1], and the Baldrige health care criteria for performance excellence [7] have been used as sources for secondary data. The primary data are gathered through a structured questionnaire that was initially developed based on these secondary data. The responses are gathered on a five-point Likert scale [14,15]. This questionnaire then has been modified using factor analysis and validated. The primary data for this purpose were collected from the sample health care organizations in India and Iran. The primary data were further analysed for the second objective using analysis of variance (ANOVA) and post-hoc Turkey test. The results so obtained are compared, with that of the ten American health care organizations, which have received the Malcolm Baldrige National Quality Award during the period 2002-2009.

### Analysis

Using factor analysis, a model has been developed and validated for measuring quality and performance in health care organizations. A null hypothesis that "India and Iran are not different in practicing the philosophy of total quality management for performance excellence in health care" is tested using the analysis of variance

## Results

The means indicate that privately run organizations have been the best followed by the government ones in the context of non-financial performance, patient focus, workforce and process, and work environment. Government organizations are found lagging behind the other two categories in leadership and quality planning. The results related to goal setting and communication show a mixed pattern. A post-hoc Turkey test [16] is used to compare the three types of health care organizations with each other (two at a time). The p-values so obtained indicate that the private and semi government hospitals are significantly different in patient focus, whereas, the leadership aspect is found significant when private hospitals are compared with the government ones. Comparison between the government and semi-government hospitals did not show any significant difference between them.

The null hypothesis of equal means among the three types of health care services is also tested for the two countries separately. The ANOVA indicates that the three types are significantly different in India on knowledge management (p = .032) with the government services being the best followed by the semi-government set ups. In Iran it is the leadership that makes a significant difference (p = .010) among the three types. Private services have got the best mean on this construct.

Using factor analysis, a model has been developed and validated for measuring quality and performance in health care organizations. The model is referred to as instrument for health care quality and performance measurement. The instrument consists of ten constructs, namely, nonfinancial performance, patient focus, quality planning, workforce and process, goal setting, leadership, work environment, communication, knowledge management, and financial performance. The constructs are then compared with the Baldrige framework [7], a guide suggested by the American Hospital Association [1], and the background document of the

WHO European conference (2008) on health systems [2]. The contents of the instrument are also verified with one of the seminal studies using the Malcolm Baldrige national quality award for comparing quality practices in different countries [16]. The constructs are found matching with the standards referred above and taking care of all major requirements outlined for health care performance systems (Table 1).

**Table 1:** Differences of two countries in case of ten criteria

		Sum of squares	Df	Mean square	F	**Sig**.
**NFP**	Between Groups	0.272	2	0.136	0.164	.849
	
	Within Groups	88.547	107	0.828		
	
	Total	88.818	109			

**PF**	Between Groups	3.816	2	1.908	2.864	.061
	
	Within Groups	71.280	107	0.666		
	
	Total	75.097	109			

**QP**	Between Groups	0.333	2	0.171	0.313	.732
	
	Within Groups	58.513	107	0.547		
	
	Total	58.856	109			

**WFP**	Between Groups	2.676	2	1.338	2.435	.092
	
	Within Groups	58.797	107	0.550		
	
	Total	61.474	109			

**GS**	Between Groups	1.069		0.534	1.059	.351
	
	Within Groups	54.024	107	0.505		
	
	Total	55.093	109	1.500		

**LD**	Between Groups	3.000	2		2.971	.055
	
	Within Groups	54.026	107	0.505		
	
	Total	57.027	109			

**WE**	Between Groups	0.285	2	0.142	0.277	.758
	
	Within Groups	55.010	107	0.514		
	
	Total	55.295	109			

**CMN**	Between Groups	1.280	2	0.640	1.050	.354
	
	Within Groups	65.265	107	0.610		
	
	Total	66.545	109			

**KM**	Between Groups	2.645	2	1.323	1.881	.157
	
	Within Groups	75.227	107	0.703		
	
	Total	77.873	109			

**FP**	Between Groups	0.367	2	0.184	0.135	.874
	
	Within Groups	145.233	107	1.357		
	
	Total	145.600	109			

## Discussion

All the ten dimensions of quality and performance correlate significantly with each other. Among the strong correlations are quality planning--workforce and process, patient focus--workforce and process, and communication--work environment. Non-financial performance has got relatively better relationship with communication, patient focus, and financial performance. The leadership- non-financial performance correlation has been comparatively lower than that with financial performance. The study by Schniederjans et al. [16], involving manufacturing, processing, and service companies, has also got significant correlations among all the nine dimensions, they have evolved. A null hypothesis that "India and Iran are not different in practicing the philosophy of total quality management for performance excellence in health care" is tested using the analysis of variance. Except for goal setting and work environment the F-values did not show any significant difference between the two populations. The mean values on the ten constructs for Indian hospitals exhibit the following hierarchy of the constructs in order of their decreasing importance- work environment, leadership, goal setting, patient focus, knowledge management, quality planning, financial performance, workforce and process, non-financial performance, communication. In case of Iran, this hierarchy appears as following- non-financial performance, patient focus, work environment, knowledge management, communication, financial performance, leadership, quality planning, workforce and process, and goal setting. Comparative analyses of the means of the average scores on the ten constructs are also conducted by size and type of the responding organizations. The ANOVA indicates that the whether a hospital is private, semi-government, or government, it does not have any significant effect on its perception and assessment about the quality measures. A post-hoc Turkey test, comparing the three types of health care organizations with each other indicates that the private and semi-government hospitals are significantly different in patient focus, whereas, the leadership aspect is found significant when private hospitals are compared with the government ones. Comparison between the government and semi-government hospitals did not show any significant difference between them. Analysis of variance is also conducted to test the null hypothesis of equal means among the three types of health care services considering the two countries separately.

## Conclusion

Finally it has been found that the three types of hospitals are significantly different in India on knowledge management with the government services being the best followed by the semigovernment set ups. In Iran it is the leadership that makes a significant difference among the three types. Private services in Iran have got the best score on this construct. Keeping the theme of the thesis in mind, the perceptions and assessments of the Indian and Iranian hospitals on TQM are benchmarked against the performance of those hospitals in the USA which have received the Malcolm Baldrige National Quality Award in the health care sector. The average scores of Indian and Iranian hospitals on different constructs of the IHCQPM model are compared with the major results achieved by the recipients of the MBNQ award. In no case the hospitals from India and Iran are found scoring close to the benchmarks.

Health care organizations are supposed to be more customer-oriented than all other organizations owing to the nature of service they are meant to offer. The quality of their services is crucial to the patients and the community. Regular surveys of satisfaction from all the stakeholders as well as the employees need to be conducted to continually assess, monitor, and improve the performance.

## Competing interests

The authors declare that they have no competing interests.

## Authors' contributions

AHG and JF contributed to the study design. Data acquisition was carried out by AHG contributed to data analysis. JF was supervisor of the thesis and revised the manuscript. All authors read and approved the final version of the manuscript.
